# Supplementary material for: WordCommentsAnalyzer: A windows software tool for qualitative research
Source: F1000Res. 2018 Sep 4;7:536. Originally published 2018 May 3. [Version 2] doi: 10.12688/f1000research.14819.2 (PMC6137411; doi:10.12688/f1000research.14819.2)
Supplement: Supplementary file 3 [file f1000research-7-17614-s0002.tgz › b2a79496-4ada-47c4-9682-a99ce867836d.zip!/Eight Iranian Health Journals/Archives of Rehabilitation_52.docx]

© Archives of Rehabilitation. This work is licensed under CC BY-NC 4.0 (https://creativecommons.org/licenses/by-nc/4.0/).

Volume & Issue: دوره 7 - شماره 4

Title: آسیب‌شناسی روانی و مشکلات شخصی و ارتباطی مردانی که مرتکب خشونت خانوادگی می‌شوند

http://rehabilitationj.uswr.ac.ir/article-1-18-fa.html

هدف: این پژوهش با هدف بررسی آسیب شناسی روانی و مشکلات شخصی و ارتباطی مردانی که مرتکب خشونت خانوادگی می‌شوند انجام یافت. روش بررسی: مطالعه حاضر از نوع مطالعات مقطعی است که در آن 230 مرد متأهل که با استفاده از روش نمونه گیری خوشه‌ای چند مرحله‌ای از مراکز مختلف مانند سینماها، گردشگاه‌ها، دادگاه‌های خانواده، پارک ها، فرهنگ سراها (این مراکز به گونه تصادفی از نواحی 2، 5، 12 و 18 شهر تهران در طرح پژوهش قرار گرفته بودند) انتخاب شده بودند، پس از آگاهی از اهداف پژوهش و اعلام رضایت در پژوهش شرکت کردند. داده‌های پژوهش از طریق مقیـاس تاکتیک های تعارضی برای شناسایی گروه مرتکبین و غیر مرتکبین خشونت خانوادگی جمع آوری شد. سپس با استفاده از پرسشنامه نیمرخ شخصی و ارتباطی، و فهرست تجدید نظر شده علائم، آسیب شناسی روانی و مشکلات شخصی و ارتباطی همراه خشونت مورد بررسی قرار گرفت. یافته‌ها: نتایج پژوهش تفاوتهای معناداری را برای گروه‌های مرتکب و غیرمرتکب خشونت در همه مقیاس‌های پرسشنامه نیمرخ شخصی و ارتباطی غیر از خرده مقیاس حسادت نشان داد (0/05&geP) و بر همین اساس هر دو گروه در همه مقیاس‌های فهرست تجدید نظر شده 90 آیتمی علائم، تفاوت معنادار آماری نشان دادند (0/05&geP). نتیجه‌گیری: مردانی که مرتکب خشونت خانوادگی و همسرآزاری می‌شوند از آسیب شناسی روانی و مشکلات شخصی و ارتباطی بسیار بیشتری در مقایسه با مردانی که مرتکب خشونت نمی‌شوند رنج می‌برند.

Title: نقش توان‌بخشی شناختی در کاهش نقایص کارکردهای اجرایی و نشانه‌های وسواسی-اجباری بیماران اسکیزو-وسواسی

http://rehabilitationj.uswr.ac.ir/article-1-19-fa.html

هدف: کارکرد شناختی ضعیف‌تر بیماران اسکیزو-وسواسی در پژوهش‌های متعددی یافته شده است. نظریه «آسیب شناسی افزوده» بیان می‌کند که اشکالات شناختی اختلال وسواسی - اجباری در بیماران اسکیزو-وسواسی به اشکالات شناختی اسکیزوفرنیا افزوده می‌شود. بر این اساس هدف از این پژوهش پاسخ به این پرسش است که آیا بین نشانه‌های وسواسی-اجباری و نقائص کارکردهای اجرایی رابطه متقابلی وجود دارد به نحوی که تغییر در یکی منجر به تغییر در دیگری گردد؟ روش بررسی: در این جهت در یک طرح پژوهش تک موردی با استفاده از خط پایه چندگانه، 4 بیمار اسکیزو-وسواسی با انتخاب هدفمند گزینش و تحت توانبخشی شناختی معطوف به بهبود کارکردهای اجرایی قرار گرفتند و سیر تغییرات شناختی و بالینی آنها بررسی گردید. یافته‌ها: نتایج نشان داد که توانبخشی شناختی موجب کاهش نشانه‌های وسواسی-اجباری در حداقل دو تن از آزمودنی‌ها و بهبود کارکردهای اجرایی در آنها گردید و به نظر می‌رسد بین میزان بهبودی در کارکردهای اجرایی و کاهش نشانه‌های وسواسی-اجباری رابطه مستقیمی وجود دارد. نتیجه‌گیری: بدین ترتیب نتیجه گرفته می‌شود که فرضیه تعامل بین نشانه‌های وسواسی-اجباری با کارکردهای اجرایی را می‌توان مورد تأیید قرار داد.

Title: بررسی بیومکانیکی ترمیم خود به خودی آسیب‌های غضروفی-استخوانی در زانوی خرگوش

http://rehabilitationj.uswr.ac.ir/article-1-20-fa.html

هدف: هدف از این مطالعه بررسی ترمیم خود به خودی آسیب‌های 4×5 میلیمتر در غضروف شیار پاتلار خرگوش نر بالغ است. روش بررسی: در یک مطالعه تجربی و مداخله‌ای21 خرگوش نر بالغ سفید از نژاد Dutch (وزن 0/2 کیلوگرم±2 کیلوگرم، 4 ماهه) برای این مطالعه انتخاب شدند. خرگوش‌ها به گونه تصادفی، به 3 گروه (4، 8 و 16 هفته) تقسیم شدند. در هر گروه بین 6-8 خرگوش قرار داده شد. پای مقابل به عنوان کنترل انتخاب شد. در گروه آزمایشی، در شرایط استریل و بیهوشی جراحی ناحیه زانو انجام شد و نقصی به قطر 5 و عمق 4 میلیمتر در شیار پاتلار استخوان ران توسط دریل ایجاد شد. پای مقابل بدون جراحی جهت مقایسه استفاده شد. پس از گذشت چند هفته خرگوش‌های هر گروه کشته شده و هر دو زانوی آنها جدا شد و به وسیله آزمایش‌های بیومکانیکی با روش ایندنتاسیون (indentation) مورد ارزیابی قرارگرفتند. ضخامت غضروف مادول الاستیک آنی و تعادلی پس از 900 ثانیه اندازه‌گیری شد. یافته‌ها: نتایج نشان داد که هیچ اختلاف معنی‌داری درمیانگین مقدار مادول الاستیک آنی و تعادلی بین هفته‌های (4، 8 و 16) در هر دو پا وجود ندارد. از سوی دیگر در مقایسه بین دو پا مشخص شد که مادول الاستیک آنی در 16 هفته بین دو پا دارای اختلاف معنی‌داری است، به گونه‌ای که میانگین مقدار این متغیر در پای آسیب دیده بیشتر از پای سالم بود. نتیجه‌گیری: نتایج نشان‌ داد که آسیب‌های غضروفی- استخوانی با اندازه‌ی 4×5 میلیمتر، در شیار پاتلار خرگوش نر بالغ به گونه خود به خود ترمیم می‌شود.

Title: بررسی نگرش کارکنان مراکز جامع توان‌بخشی بهزیستی استان تهران نسبت به وضعیت عوامل درون سازمانی استرس‌زا و شدت استرس‌زایی آنها

http://rehabilitationj.uswr.ac.ir/article-1-21-fa.html

هدف: افرادی که خدمات توانبخشی ارائه می کنند به دلیل ارتباط مستمر با مردمی که هر یک به نوعی دچار نقص، ناتوانی و مشکلات همراه آن می باشند، در معرض استرس شغلی مداوم هستند. حال چنانچه با سیاستها و فرآیندهای نامناسب سازمانی نیز مواجه شوند، استرس مضاعف حاصله می تواند بهداشت روانی وسلامت جسمانی آنان را خدشه دار نماید. هدف این مطالعه بررسی نگرش کارکنان این مراکز نسبت به وضعیت عوامل درون سازمانی استرس زا و شدت استرس زائی آنهاست. روش بررسی: در یک مطالعه توصیفی جهت تعیین وضعیت سیاستها و فرآیندهای نامناسب استرس‌زای سازمانی و شدت استرس‌زائی هر یک از این عوامل از دیدگاه پرسنل مراکز جامع توانبخشی تحت پوشش بهزیستی استان تهران، از همه کارکنان درخواست شد پرسشنامه‌ای شامل 32 سئوال را تکمیل کنند. اعتبار محتوایی پرسشنامه به تأیید صاحبنظران رسیده و پایایی آن 0/83 محاسبه شد. یافته‌ها: بر اساس نتایج بدست آمده، کارکنان بیش از همه با سیاست «ارزیابی غیر واقعی کارکنان» (%98) و فرآیند «نقص معیارهای سنجش عملکرد کارکنان» (%95) روبرو بودند و همچنین در مواجهه با سیاست «عدم تساوی حقوق و مزایای افراد همطراز»(میانگین شدت استرس=2/64 از 4) و فرآیند «نقص معیارهای سنجش عملکرد کارکنان» (میانگین شدت استرس=1/71 از 4) احساس استرس بیشتری می‌کردند. نتیجه‌گیری: با توجه به نتایج پژوهش به منظور کاهش استرس پرسنل، بعنوان اولین قدم براصلاح نظام ارزیابی و تدوین معیارهای صحیح برای سنجش عملکرد کارکنان، و اصلاح نظام پرداختها تأکید می‌شود.

Title: تعیین نیاز های توان‌بخشی صدمات ارتوپدی در زلزله بم

http://rehabilitationj.uswr.ac.ir/article-1-22-fa.html

هدف: هدف از بررسی حاضر تعیین نوع و میزان نیازهای توانبخشی مصدومین زلزله شهرستان بم در سال 1382 بوده است. روش بررسی: این پژوهش یک مطالعه مقطعی است که برروی 3000 نفر از مجروحین زلزله بم که به بیمارستان‌های کشور ارجاع شده بودند، انجام شده است. نمونه گیری از بین بیمارستان‌های کشور به صورت تصادفی ساده صورت گرفت و کلیه بیماران چهار بیمارستان منتخب به صورت سرشماری در مطالعه شرکت داده شدند. یافته‌ها: بررسی ضایعات نشان داد که 67/74 درصد ضایعات، شکستگی و دررفتگی بوده است. پس از آن ضایعات تروماتیک سیستم اعصاب مرکزی و محیطی به ترتیب 11/15 و 9/53 درصد شیوع داشت. سایر موارد مشتمل بر ضایعات بافت نرم (3/17 درصد)، زخم باز (9/48 درصد)، ضایعات عروقی (0/72 درصد) و قطع عضو (0/55 درصد) از شیوع کمتری برخوردارند. نتیجه‌گیری: نتیجه کاربردی که می‌توان از این بررسی برای مدیریت بحران در زلزله گرفت این است که بیشترین متخصصین مورد نیاز در تیم پزشکی، متخصصان ارتوپدی و توانبخشی (فیزیوتراپی و ارتوپدی فنی) می‌باشند و کلینیک‌های صحرایی توانبخشی در بیمارستان‌های صحرایی بسیار مورد نیاز است تا از عواقب ناشی از عدم ارائه توانبخشی به موقع جلوگیری شود.

Title: رابطه فشار روانی با رضامندی زناشویی در والدین کودکان عقب‌مانده ذهنی

http://rehabilitationj.uswr.ac.ir/article-1-23-fa.html

هدف: این تحقیق به منظور بررسی رابطه میزان فشار روانی با رضامندی زناشویی در والدین کودکان عقب‌مانده ذهنی آموزش‌پذیر انجام شد. روش بررسی: روش پژوهش در این تحقیق مقطعی مقایسه‎ای است. نمونه‌های پژوهش شامل دو گروه 40 نفری از والدین کودکان عقب‌مانده ذهنی آموزش‌پذیر (20 پسر و 20 دختر) و والدین کودکان عادی است که به شیوه خوشه‎ای و تصادفی ساده از مناطق 1، 5، 15 و 18 آموزش و پرورش تهران برای گروه والدین کودکان عقب‌مانده ذهنی و همتاسازی با آنها برای والدین کودکان عادی، از طریق مراجعه به مدارس وابسته به سازمان کودکان استثنایی و مدارس عادی شهر تهران انتخاب شد. جهت انجام پژوهش، دو پرسشنامه منبع استرس و رضامندی زناشویی انریچ اجرا گردید. نتایج با استفاده از آزمونهای آماری تی مستقل، همبستگی پیرسون و آنالیز کوواریانس مورد تجزیه و تحلیل قرار گرفت. یافته‌ها: نتایج پژوهش نشان داد بین فشار روانی و رضامندی زناشویی والدین کودکان عقب‌مانده ذهنی آموزش‌پذیر رابطه معنی‌داری وجود دارد (P=0/001). همچنین والدین کودکان عقب‌مانده ذهنی آموزش‌پذیر فشار روانی بیشتری را نسبت به والدین کودکان عادی تحمل می‎کنند (P=0/001). در عین حال، والدین کودکان عقب‌مانده ذهنی آموزش‌پذیر نسبت به والدین کودکان عادی از رضایت زناشویی پایین تری برخوردارند (P=0/021). همچنین، نتایج نشان داد که همبستگی بین دو متغیر فشار روانی و رضامندی زناشویی والدین کودکان عقب‌مانده ذهنی آموزش‌پذیر منفی است (r=-0/57) بدین مفهوم که با افزایش فشار روانی، رضامندی زناشویی به طور معناداری کاهش می‌یابد. نتیجه‌گیری: نتایج این تحقیق مبین این نکته است که افزایش فشار روانی والدین، تحت تأثیر حضور کودک عقب‌مانده ذهنی آموزش‌پذیر، منجر به کاهش رضامندی زناشویی والدین شده است. بعبارتی، رضامندی زناشویی را می‌توان تابعی از وجود کودک عقب‌مانده ذهنی در خانواده تلقی نمود. لذا با برگزاری جلسات آموزش و مشاوره جهت والدین کودکان عقب‌مانده ذهنی، می‌توان راههای کاهش استرس را به آنها آموزش داد.

Title: مقایسه توانایی تکرار ناکلمه در کودکان لکنتی و غیرلکنتی 8-5 ساله: بررسی فرضیه اصلاح پنهان

http://rehabilitationj.uswr.ac.ir/article-1-24-fa.html

هدف: فرضیه اصلاح پنهان مشکلی اساسی لکنت را در سیستم زبانی یا به عبارت بهتر چرخه واجی فرایند تولید کلمه می‌داند و مدعی است که افراد لکنتی در فرایند تولید گفتار نسبت مطالعه حاضر به منظور سنجش فرضیه اصلاح پنهان (Covert Repair Hypothesis) یعنی بررسی چرخه واجی فرایند تولید کلمه در کودکان لکنتی انجام شده است. روش بررسی: در پژوهش حاضر که به روش تحلیلی مورد – شاهدی انجام گرفت، حدود 40 ناکلمه دو هجایی و سه هجایی طراحی و پس از بررسی مقدماتی، به 12 کودک لکنتی که با روش نمونه‌گیری هدفمند و در دسترس انتخاب شده بودند و 12 کودک غیر لکنتی که به روش جورکردن با گروه مورد انتخاب شده بودند با محدوده سنی 8-5 سال با استفاده از نرم افزار DMDX ارائه گردید و از کودک خواسته شد که بلافاصله پس از شنیدن ناکلمه آن را تکرار نماید. پس از تکرار نا کلمه زمان واکنش هر کودک در تکرار ناکلمه توسط نرم‌افزار مذکورثبت می‌گردید و تعداد خطاهای واجی و پاسخهای صحیح آنها مشخص می‌گشت. ﻳافته‌ها: نتایج حاکی از آن بود که کودکان لکنتی در کلیه موارد به استثنای زمان واکنش ناکلمه‌های سه هجایی عملکرد ضعیفتری را ارائه نمودند. البته نتایج پژوهش از نظر آماری معنا دار نبود (P=0/2). نتیجه‌گیری: شواهد حاصل از این پژوهش تفاوتهایی را میان عملکرد کودکان لکنتی و غیر لکنتی نشان می‌دهد که می‌تواند امکان وجود نقص در فرایندهای زبانی کودکان لکنتی به ویژه رمزگزاریهای واجی آنان در مکانیسم تولید واژه را مطرح نماید. البته این تفاوتها معنی دار نبودند که می‌تواند به علت کم بودن تعداد نمونه باشد. همچنین الگوی تفاوتهای بدست آمده میان دو گروه با فرضیات CRH همخوانی نشان می‌داد.

Title: مقایسه تمیز ادراک بینایی در سه گروه از دانش‌آموزان عادی، عقب‌مانده‌ ذهنی آموزش‌پذیر و دارای اختلالات یادگیری بی‌سرپرست تحت پوشش بهزیستی شهر تهران

http://rehabilitationj.uswr.ac.ir/article-1-25-fa.html

هدف: یکی از آزمونهای تشخیص نارساخوانی در کودکان، آزمون تمیز ادراک بینایی ریورسال است که به وسیله آن امکان تشخیص اختلال در دانش‎آموزان دوره‎ ابتدایی به ویژه کسانی که در هفته یا ماههای اول تحصیل هستند امکان پذیر می‎‌گردد. هدف این پژوهش، ارزیابی آزمون ریورسال و مقایسه آن در سه گروه از دانش‎آموزان 8 تا 12 ساله‎ عادی، عقب‌مانده‎ ذهنی آموزش‎پذیر و دانش‎آموزان دارای اختلالات یادگیری در خواندن (نارساخوان) می‎باشد. روش بررسی: پژوهش حاضر از نوع مطالعات مقطعی- مقایسه‌ای است که بر روی سه گروه 50 نفره از دانش‎آموزان 8 تا 12 ساله عادی، عقب‌مانده ذهنی آموزش‎پذیر و نارساخوان که با انتخاب ساده تصادفی از مراکز نگهداری کودکان بی‌سرپرست بهزیستی شهر تهران گزینش شدند انجام گردیده است. یافته‎ها: نتایج پژوهش، اختلاف معناداری را بین نمرات آزمون هر سه گروه مورد مطالعه نشان داد (P=0/001). بالاترین نمرات متعلق به گروه دانش‎آموزان عادی بود و کمترین نمرات متعلق به دانش‎آموزان عقب‌مانده ذهنی آموزش‌پذیر است. گروه دانش‎آموزان دارای اختلالات یادگیری (نارساخوان) از این نظر بین گروه دانش‎آموزان عادی و عقب‌مانده ذهنی آموزش‌پذیر قرار دارند. اختلاف نمره منفی دانش‎آموزان عقب‌مانده‎ی ذهنی آموزش‌پذیر با دانش‌آموزان عادی بیش از اختلاف نمره آنها با دانش‎آموزان نارساخوان است. همچنین این پژوهش نشان داد که نارساخوانان در ادراک بینایی دچار مشکل هستند. نتیجه‌گیری: این تحقیق نشان داد که کودکان نارساخوان در ادراک بینایی خود دچار مشکلاتی می‎باشند و همچنین بین نمرات بدست آمده از سه گروه مورد آزمایش(عقب‌مانده ذهنی آموزش‎پذیر، عادی و نارساخوان) تفاوت معنی داری وجود دارد.

Title: ارزیابی وضعیت آموزشی رشته‌های توان‌بخشی دانشگاه علوم بهزیستی و توان‌بخشی از دیدگاه دانشجویان در سال 83-1382

http://rehabilitationj.uswr.ac.ir/article-1-26-fa.html

هدف: هدف این پژوهش شناخت و ارزیابی وضعیت آموزشی رشته‌های توانبخشی دانشگاه علوم بهزیستی وتوانبخشی است. روش بررسی: این پژوهش از نوع توصیفی و با استفاده از دیدگاه دانشجویان سال سوم و چهارم کارشناسی و سال دوم کارشناسی ارشد رشته‌های توانبخشی صورت گرفته است. دراین پژوهش ازروش تمام شماری (160 ‌نفر)‌ استفاده شد. داده‌های مربوطه ازطریق پرسشنامه و با همکاری نمایندگان دوره‌های مختلف دانشجویان رشته‌های توانبخشی جمع آوری و با استفاده از مقیاس لیکرت ازطریق ضریب همبستگی واستفاده از روشهای آمار توصیفی تجزیه وتحلیل شد. یافته‌ها: یافته‌های نهایی این پژوهش نشان داد وضعیت ارزیابی برای هریک از سرفصلهای آموزشی به شرح ذیل بوده است. وضعیت محل تشکیل کلاسهای تئوری «نسبتاً مطلوب»، وضعیت محل تشکیل کلاسهای عملی «نسبتاً مطلوب» وضعیت کلاسهای عملی «نامطلوب»،‌وضعیت اداره امور کلاسها «نسبتاً مطلوب»، وضعیت کارشناسان آموزشی «نسبتاً مطلوب»، ‌وضعیت برنامه‌ریزی دروس «نامطلوب»،‌وضعیت نحوه ارائه دروس تئوری «نسبتاً مطلوب»، وضعیت آموزش درمراکز بالینی «‌نامطلوب» ‌و وضعیت مراکز بالینی « نامطلوب» می‌باشد. نتیجه‌گیری: از مجموع نظرات حاصل چنین بر می‌آید که ناهماهنگی در آموزش تئوری و بالینی وجود داشته و کمبود اساتید مجرب در آموزش تئوری و بالینی وجود دارد. برخی دانشجویان دلیل غیبت از کلاسها را تدریس نامناسب عنوان می‌کنند و ضعف دسترسی به اساتید درسی و راهنما و ضعف قوانین و مقررات آموزشی از دیگر مشکلات آنان است.

Title: تأثیر خشونت‌های جنسی در بروز اختلال استرس پس از ضربه (PTSD)

http://rehabilitationj.uswr.ac.ir/article-1-27-fa.html

خشونت خانگی به هر نوع عمل خشونت‌آمیزی که به اختلاف جنسیت مبتنی باشد گفته می‌شود. یکی از انواع خشونت که به دلیل مسائل فرهنگی و اجتماعی کمتر مورد توجه قرار گرفته، خشونت جنسی علیه کودکان و زنان است. یکی از شایع‌ترین اختلالات روانپزشکی در خصوص خشونتها و تهاجم جنسی اختلال استرس پس از ضربه می‌باشد. PTSD وقتی بروز می‌کند که شخص فشار روانی و هیجانی را که شدت آن عملاً برای هرکسی آسیب رسان خواهد بود تجربه کرده باشد. این اختلال شامل ویژگیهایی مانند یاد‌آوری خاطرات و تجربه مجدد سانحه در رویا و بیداری، اجتناب مستمر از یاد‌آوری سانحه و کرختی عاطفی و بی‌حسی و برانگیختگی شدید می‌باشد، این علائم حداقل باید یکماه تداوم یابد تا فرد مبتلا به PTSD گردد. یکی از خشونت‌های خانگی سوء رفتار جنسی و همسر آزاری است که در کودکان و زنان بصورت افسردگی، اضطراب و PTSD مشاهده می‌گردد.

Title: گزارش یک مورد بیمار مبتلا به سندرم ولفرام

http://rehabilitationj.uswr.ac.ir/article-1-28-fa.html

سندرم ولفرام عبارت است از همراهی دیابت شیرین جوانان، آتروفی عصب بینایی، دیابت بیمزه مرکزی و کری حسی عصبی و گاهی سندرم DIDMOAD نیز نامیده می‌شود این سندرم یک بیماری ارثی اتوزومال مغلوب و نادر با شیوع 1 در 770000 نفر می‌باشد. اشکال نکامل این سندرم، همچنین مواردی که دارای علائم متنوع دیگری بوده‌اند نیز گزارش شده است. سیر طبیعی سندرم ولفرام چنان است که بیشتر بیماران سرانجام دچار اکثر عوارض این اختلال نورودژنراتیو پیشرونده خواهند شد. دیابت شیرین جوانان و آتروفی عصب بینایی بهترین معیار تشخیصی سندرم ولفرام است. در این مقاله بیماری معرفی می‌شود که مبتلا به دیابت شیرین وابسته به انسولین و دیابت بیمزه مرکزی بود و در بررسی‌های بیشتر آتروفی دو طرفه عصب بینایی و اختلال شنوایی در فرکانس‌های بالا نیز تشخیص داده شد. والدین کودک نسبت خویشاوندی داشته و بیمار اولین مورد مبتلا در خانواده است.
